# Assessing Gluten-Free Soy Bread Quality and Amino Acid Content

**DOI:** 10.3390/foods12061195

**Published:** 2023-03-12

**Authors:** Teruyo Nakatani, Manami Tanaka

**Affiliations:** Department of Food Science and Nutrition School of Food Science and Nutrition, Mukogawa Women’s University, Nishinomiya 663-8558, Hyogo, Japan

**Keywords:** protein, amino acids, soy, gluten-free rice flour bread

## Abstract

The nutritional and palatability relevance of bread prepared with soy flour was examined. There are a few effective nutritional measures that combine palatability, convenience, and functionality in the suppression of muscle loss (contributing to the improvement and prevention of sarcopenia). Therefore, in the present study, we attempted to produce bread using soybeans, which are rich in amino acids involved in the synthesis and degradation of skeletal muscle proteins. Rice flour was also used to avoid gluten intolerance. The bread was baked in an automatic bread maker, and the rheological properties of its breadcrumbs were determined using a creep meter. We found that a 70 g slice of soy bread satisfied approximately one-fifth of the daily nutritional requirement for leucine. Although soy decreased the specific volume of bread by preventing starch construction, the use of preprocessed rice flour recovered the volume, and corn starch improved the taste. We propose that the addition of soy bread to the daily diet may be an effective protein source.

## 1. Introduction

The 2022 annual report of the World Health Organization (WHO) shows that both healthy life expectancy (HALE) and overall life expectancy (LE) have increased in developed countries [1]. HALE was defined by the WHO in 2000 as the average period during which a person experiences no impediment to everyday life. The time difference between LE and HALE represents the “unhealthy period”, during which there are limitations to everyday life (such as being bedridden), thereby leading to a deterioration of an individual’s quality of life and a reduction in the social security burden. Therefore, an increasing gap between HALE and LE has raised concerns. For instance, in Japan, an approximately 10-year gap exists; thus, strategies are needed to extend HALE.

Sarcopenia is defined as an age-related decline in skeletal muscle mass and physical function (strength) and is associated with an increased risk of falling and disability [2,3,4,5,6]. To acquire a super-aged society, the need to establish preventive measures against sarcopenia increases. This involves maintaining or increasing skeletal muscle mass, requiring the stimulation of protein synthesis and the suppression of proteolysis in skeletal muscle tissue. However, efficient prophylaxes to minimize the sarcopenia burden through diet regulation have not yet been sufficiently developed. Particularly for the elderly, nutrition is important to prevent the development and progression of skeletal muscle loss. Notably, aged muscles require more amino acids to stimulate their anabolism due to a reduction in muscle protein synthesis [7], known as anabolic resistance. However, the inability of skeletal muscles to respond to low doses of essential amino acids has been reported with aging, whereas higher doses are capable of stimulating muscle protein synthesis to a level that is equal to that of young individuals [8]. However, appropriate protein intake remains essential for preventing the development of sarcopenia, and, with the tendency that elderly people decrease their food intake, it is difficult to ensure adequate protein consumption. Therefore, efficient protein ingestion is a key factor contributing to the stimulation of protein synthesis and the suppression of proteolysis in the skeletal muscles of elderly people.

Certain amino acids play a more prominent role in protein metabolism, rendering them ideal targets for inclusion in a protein-rich food source. The mechanistic/mammalian target of rapamycin (mTOR) is an evolutionarily conserved serine/threonine kinase that is known to be a master regulator of cellular metabolism. mTOR stimulates anabolic processes, such as protein synthesis while simultaneously inhibiting autophagy, which includes protein degradation. Leucine, a branched-chain amino acid (BCAA), activates mTOR signaling more efficiently than other amino acids [9]. In addition, lysine suppresses protein degradation through autophagy inhibition [10,11]. Soy protein has high-protein digestibility-corrected amino acid and digestible indispensable amino acid scores, which consider digestibility and absorption capacity [12]. Nutritionally, soy possesses complete, high-quality proteins containing essential amino acids rich in BCAAs (comprising approximately 20% of amino acids in boiled soybean) and approximately 7% lysine. Additionally, the predominant soy protein, glycinin, contains a sequence similar to that of the ubiquitin ligase CbI-b inhibitor peptide, whereby it produces an inhibitory effect on CbI-b-mediated skeletal muscle atrophy in vitro and in vivo [13,14]. Indeed, dietary soy protein upregulates skeletal muscle volume and strength in humans with low physical activity or those that are bedridden [15].

Another important factor affecting efficient protein synthesis is the timing of its intake, which can influence muscle hypertrophy. In particular, a high-protein (BCAA) breakfast is more efficient in establishing muscular hypertrophy than an equal distribution of protein intake across the day or a higher intake at dinner. This time-dependent effect is affected by clock genes, such as *Clock* and *Bmal1,* and by BCAA ingestion [16]. However, there is a tendency for people to consume less protein during breakfast [17].

The current study seeks to evaluate formulations for producing protein-rich bread that can assist in efficient protein synthesis, which in turn can prevent the loss of skeletal muscle mass. Although wheat-based bread is commonly consumed for breakfast, it contains gluten that can induce gastrointestinal discomfort. Zonulin is a physiological modulator of intestinal tight junctions that leads to a leaky gut. Gliadin, a protein component of wheat gluten, activates zonulin signaling, which increases intestinal permeability to macromolecules [18,19,20,21,22]. Therefore, taking gluten intolerance into account, we used rice flour as a grain product that can be consumed safely over a long period. To achieve a positive nutritional effect, consistent consumption is important, and we evaluated the quality of the bread produced.

The specific aims of the study were to investigate the effects of sub-ingredients on the quality of bread baked with rice flour and soy flour supplements as an initiative to increase protein consumption by humans and thus contribute to the prevention of sarcopenia. Our results showed that although soy flour supplementation to rice bread reduced the specific volume (bread quality indicator), the volume was recovered using preprocessed rice flour and cornstarch, which participate in the fermentation process.

## 2. Materials and Methods

### 2.1. Ingredients

The ingredients of the bread comprised rice flour (Mizuhochikara; Kumamoto Flour Milling Co., Ltd. Kumamoto, Japan), soy flour (Perican Co., Ltd., Saitama, Japan), wheat flour (Nisshin Flour Milling Inc., Tokyo, Japan), cornstarch (Tokan Co., Ltd., Aichi, Japan), starch (Imazu Co., Ltd., Osaka, Japan), sugar (Mitsui Sugar Co., Ltd., Tokyo, Japan), salt (Naikai Salt Industries Co., Ltd., Okayama, Japan), preprocessed rice flour (Musubi Co., Wakayama, Japan), dry yeast (Nisshin Flour Milling Inc., Tokyo, Japan), and canola oil (The Nisshin OilliO Group Ltd., Tokyo, Japan).

### 2.2. Formulations and Baking

The bread samples were prepared using an original method designed by us. The components of each base formulation (baker’s %) were rice flour (100), water (82), sugar (5), salt (2.1), dry yeast (1.5), and canola oil (5.7). For wheat bread, wheat flour was used instead of rice flour. The total amount of flour was adjusted when soy flour, preprocessed rice, and cornstarch were added to the formula. The bread was baked using the rice bread program or wheat bread program of an automatic bread machine (ST-MT3; Panasonic Corp., Osaka, Japan). The baked bread loaves were cooled at room temperature (25 ± 2 °C) for 1 h before performing various measurements.

### 2.3. Specific Volume Measurements

After cooling, the weight and volume of the bread loaves were measured. The volume was determined based on the rapeseed displacement method (AACC Method 10-05.01; AACC, 2000). The mass was measured using a digital scale (TANITA CORPORATION). The specific volume was calculated as the ratio of volume to weight (mL/g).

### 2.4. Analysis of Rheological Properties

A creep meter (Rheoner RE2-33005S; Yamaden Co., Ltd., Tokyo, Japan) was used to determine the rheological properties of the breadcrumbs. The parameters for measuring rupture characteristics included sample size, 30 × 30 × 20 mm; plunger, wedge form (No. 49, Yamaden, with a base width of 1 mm and length of 30 mm); measurement strain rate, 100%; and compression speed, 1.0 mm/s. Conditions for the measurement of bread texture included sample size, 30 × 30 × 20 mm; plunger, circular form (12 mm diameter); measurement strain rate, 50%; and compression speed, 5 mm/s. Each experiment was performed at least in triplicate, and averages were obtained for the data.

### 2.5. Analysis of Protein and Amino Acid Content

Protein and amino acid contents of bread types (wheat, rice, and soy bread) were analyzed using the combustion method and HPLC (JLC-500/v2; JEOL. Ltd., Tokyo, Japan), respectively, by the Japan Food Research Laboratories. For each bread sample, 0.31–0.39 g and 0.2–0.3 g were used for the protein and amino acid analyses, respectively. The samples were dissolved in 20 mL of 20% hydrochloric acid containing 0.04% 2-mercaptoethanol. After hydrolysis (110 °C, 24 h), the samples were quantitated and prepared for HPLC measurement. Ninhydrin was used as the reaction reagent, and tryptophan was measured by HPLC (fluorescence detection), whereas other amino acids were measured by post-column derivatization. Each sample was analyzed twice.

### 2.6. Measurement of Water Absorption

The water absorption of each flour sample was determined using the technique developed by Matsuki et al. [23]. Seven holes (1.5 mm diameter) were created in the bottom of a column-shaped plastic container (4.7 cm × 9 cm × 1.5 mm), 12 mm apart. One piece of glass fiber filter paper (GA-55, 4.7 cm diameter, Advantec Tokyo Roshi, Tokyo, Japan) was placed inside the container. The flour sample (10 g) was weighted in the container. A weight (25 g) was placed onto the container to ensure that the sample did not float during the experiment. The container was placed in a tray filled with 1 cm-deep water to let water in through the holes at the bottom. A piece of filter paper (#2, 7.0 cm diameter, Advantec Tokyo Roshi, Tokyo, Japan) was placed under the container in the tray to avoid tight contact between the bottom of the container and the tray. The container was taken out of the water for weighing and was quickly placed back in the water. The amount of water in the flour sample was calculated from the moisture content of the flour.

### 2.7. Sensory Analysis

Sensory analysis of the bread was carried out according to Japanese Industrial Standard (JIS; 9080:2004) [24] by a panel of 30 females, aged 21–22 years. Sensory attributes included appearance, aroma, taste, texture, sponginess, stickiness, chewiness, and firmness. A ten-point hedonic scale was applied to evaluate each sensory attribute. Panelists scored on a scale from 1 (disliked extremely) to 10 (liked extremely). All sessions were performed in single booths in an air-conditioned room at 20–22 °C. Before the first and between each sensory sample, the panelists rinsed their mouths (≥ 10 s) with water. Approximately 10 g of each sample was provided (3 × 3 × 3 cm) at the same time to each panelist, 2 h after baking. The sensory profiles of the optimized soy rice bread with preprocessed rice flour and/or cornstarch were assessed, and comparisons were made with reference to the soy rice bread. The test was performed with prior approval from the Ethics Committee for Research with human beings (No.22-05).

### 2.8. Statistical Analysis

All results were obtained from at least three separate experiments. Statistical differences were analyzed via one-way analysis of variance (ANOVA) and Tukey’s range test for multiple comparisons using IBM SPSS (version 26; IBM Japan, Ltd., Tokyo, Japan). The results are expressed as mean ± standard deviation (SD), and *p* < 0.05 was considered statistically significant.

## 3. Results and Discussion

### 3.1. Replacement Rate of Soy Powder

**Figure 1:**
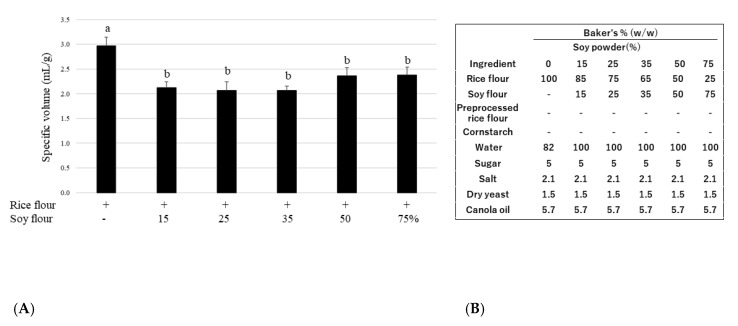
(**A**) Effect of soy flour substitution amount on the specific volume of bread; (**B**) Recipes for soy rice bread. Data are shown as mean ± SD. Different letters indicate *p* < 0.05 between groups.

To determine the ideal amount of soy flour for baking rice bread, we investigated 15, 25, 35, 50, and 75% replacements of soy flour with rice flour. The specific volume is an important indicator of the technological quality of bread and is used to express the technological aptitude of a formulation for bread production [25]. With soy, the specific volume significantly decreased when compared with that of the control (0% soy). Although the specific volume of loaves did not differ significantly among the soy groups (Figure 1), a 35% soy replacement bread was selected as a strong beany flavor was noted at soy contents > 50%.

### 3.2. Changes in Bread-Specific Volume after Soy Addition

Saito et al. [26] reported that the addition of hot water (at approximately 70 °C) to the bread batter led to the swelling of the starch grains, which began to string together, resulting in superior bread quality (highest specific volume and soft texture) when compared with those obtained at other temperatures. We examined how auxiliary ingredients could improve the reduction in the specific volume via the addition of soy to rice flour bread as a preliminary experiment and found that the addition of hot water (70 °C) or pregelatinized rice flour was the most efficient (data not shown). This was likely due to the pregelatinization effect of starch; therefore, we used pregelatinized rice flour.

**Figure 2:**
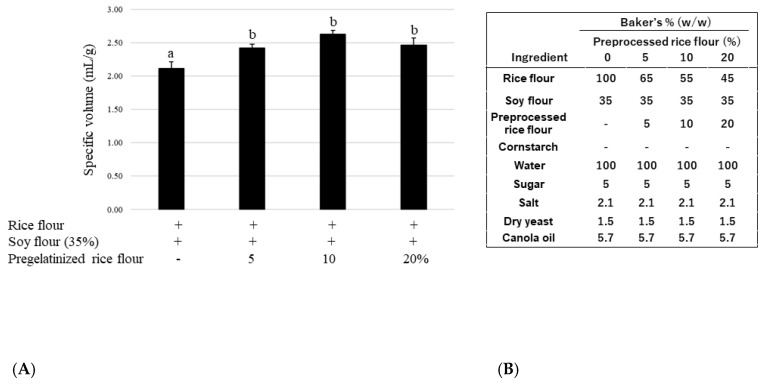
(**A**) Effect of soy flour substitution amount on the specific volume of bread; (**B**) Recipes for soy rice bread with preprocessed rice flour. Data are shown as mean ± SD. Different letters indicate *p* < 0.05 between groups.

**Figure 3:**
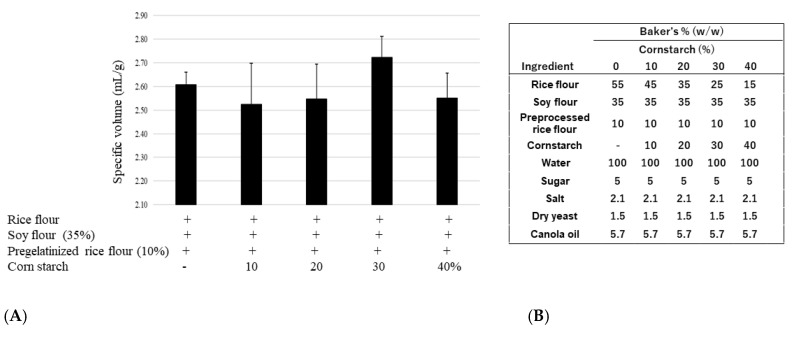
(**A**) Effect of pregelatinized rice flour substitution amount on specific volume; (**B**) Recipes for soy rice bread with preprocessed rice flour and cornstarch.

Next, the most appropriate quantity of pregelatinized rice flour was determined. Figure 2 shows that the specific volume of the bread significantly increased with increased ratios of pregelatinized rice flour. Although there were no statistical differences between 5, 10, and 20% (ratio to total flour) pregelatinized rice flour additions, a 10% pregelatinized rice flour addition produced the greatest bread volume increase; however, the bread acquired a sticky texture. To investigate potential improvement, we examined the effect of using cornstarch. Figure 3 shows that a 30% (ratio to total flour) cornstarch addition tended to improve the specific volume of the bread; however, this difference was not statistically significant.

**Figure 4:**
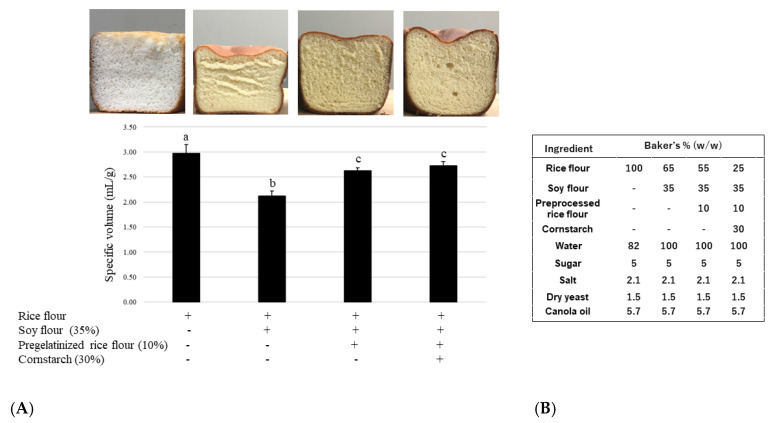
(**A**) Improvement in bread specific volume via addition of pregelatinized rice flour and cornstarch; (**B**) Recipes for soy rice bread with preprocessed rice flour and cornstarch. Data are shown as mean ± SD. Different letters indicate *p* < 0.05 between groups.

As shown in Figure 4, although the addition of soy decreased the specific volume of bread to approximately 70% in comparison to that of the control (only rice flour), the reduction was significantly recovered to approximately 90% by adding pregelatinized rice flour and to 92% by adding a combination of pregelatinized rice flour and cornstarch. There was no significant difference between the results of these two conditions; however, the sliced end of the bread gained a smoother texture (Figure 4).

### 3.3. Amino Acid Content of Bread

**Figure 5:**
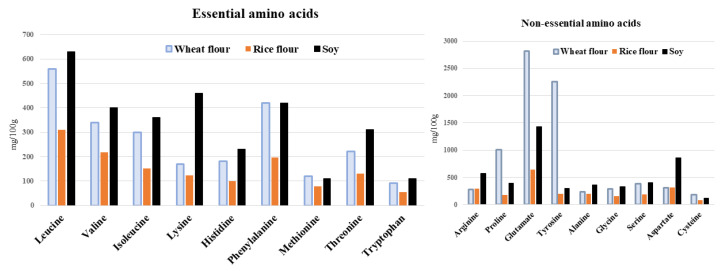
(**A**) Essential amino acid contents of different bread types; (**B**) Non-essential amino contents of different bread types.

The amino acid content of the different bread types is shown in Figure 5. Soy bread exhibited higher levels of BCAAs and lysine, whereas wheat bread contained an abundance of non-essential amino acids. BCAA levels in soy bread were approximately 1.2 and 2.0 times higher than those of wheat and rice bread, respectively (Table 1). One 70 g slice of soy bread provided approximately 19% of the BCAA, 19% of the leucine, and 18% of the lysine daily requirements (for 60 kg of body weight, according to a report from the WHO/the Food and Agriculture Organization (FAO)/United) [27,28]. Although the protein contents did not differ between wheat bread and soy bread, wheat bread contained higher non-essential amino acid contents, such as glutamic acid or tyrosine (Figure 5, Table 1). A nutritional evaluation of soy was performed using wheat flour bread [29], but rice flour breads were investigated for bread quality [30,31,32]. We analyzed not only proteins but also amino acids (which may be mainly involved in skeletal muscle metabolism). The BCAA content of a slice of soybean bread presented here was 19% of the daily recommended intake, which may not be excessive, but we highlight that it is important to consume a good balance of foods to prevent nutritional imbalance.

### 3.4. Bread Texture Properties

**Figure 6:**
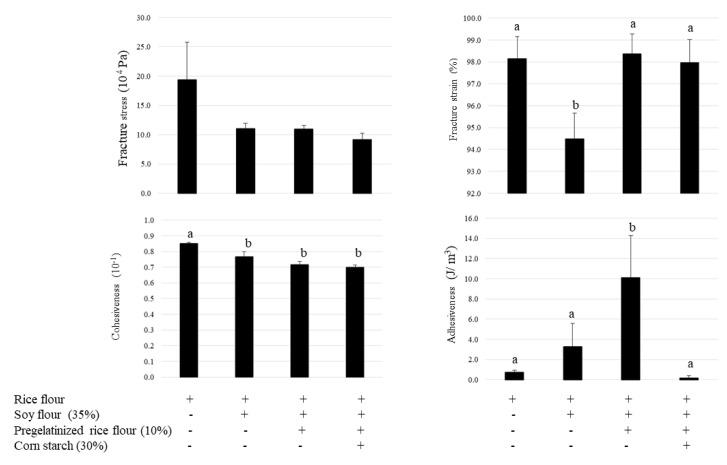
Effect of pregelatinized rice flour and cornstarch on bread texture. Data are shown as mean ± SD. Different letters indicate *p* < 0.05 between groups.

The effects of pregelatinized rice flour and cornstarch on the baking properties of soy bread are shown in Figure 6. Although the breadcrumb fracture strain was significantly lower in the bread containing soy flour than in the control, this reduction was reversed by adding pregelatinized rice flour. Cohesiveness significantly decreased in the bread containing soy flour when compared with the control, and no significant difference was observed following the addition of pregelatinized rice flour. The fracture stress tended to decrease in soy bread. Although pregelatinized rice flour significantly increased crumb adhesiveness, cornstarch returned it to the levels observed for the rice flour control. These results indicate that, although the bread attained a brittle structure when soy was used as an ingredient, the addition of pregelatinized rice flour reversed this effect by providing viscoelasticity. Cornstarch improved the excess viscosity of pregelatinized rice flour further to retain elasticity.

### 3.5. Effect of Pregelatinized Rice and Cornstarch on Soy Bread Quality

**Figure 7:**
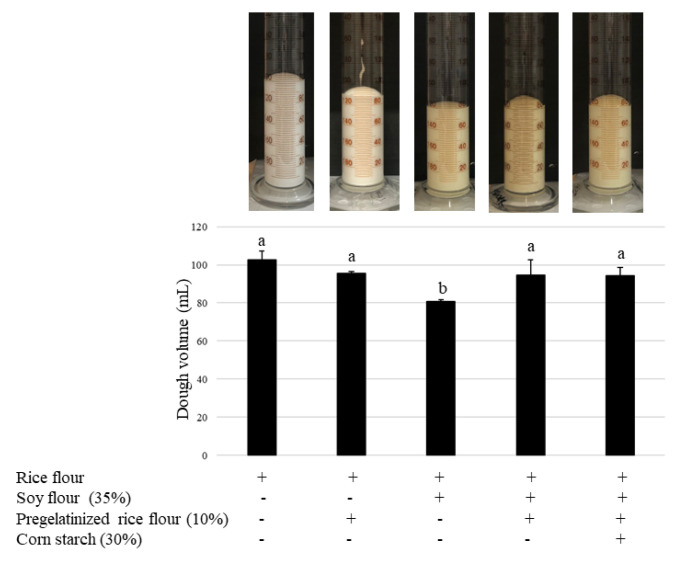
Effect of pregelatinized rice flour and cornstarch on the fermentation process (30 min). Data are shown as mean ± SD. Different letters indicate *p* < 0.05 between groups.

The volume and specific volume of bread depend on the retention of gas by the matrix during fermentation and affect bread quality [33]. Next, we examined whether the effect of specific volume recovery by pregelatinized rice and cornstarch was due to participation in the fermentation process. After 30 min of fermentation, the batter volume was lower than that of the control when using soy, but this volume was significantly recovered after adding pregelatinized rice. Cornstarch had the same effect as pregelatinized rice. In the absence of soy, pregelatinized rice did not have this effect on the batter volume (Figure 7). These results suggest that the specific volume of bread recovery observed following the addition of pregelatinized rice was due to its involvement in the fermentation process. Aoki et al. [34] assessed rice bread using different rice flour samples containing amylose contents ranging from 9.6 to 22.3% and found that the amylose content was positively correlated with the dough volume and the specific volume during leavening, which indicates that amylose plays an important role in making bread with high loaf volume. They also found that there was no correlation between the protein content and the specific volume. In this study, we used a rice flour cultivar, Mizuhochikara, which has been reported to have a high amylose content (22.3%) and low damaged starch (about 3%) when compared with other rice flour cultivars [34]. Yano et al. [35] reported that gluten-free rice bread has a high specific volume without additives, whereas rice flour bread exhibits low starch damage (<5 g/100 g). Damaged starch granules had higher water absorption than intact starch granules [36], which negatively affected bread quality, such as specific volume [37,38,39,40,41,42]. Pregelatinized rice can improve dough properties through increased cohesion, elasticity, and viscosity, which increases CO_2_ gas retention [43]. Consistent with other studies, the addition of soy flour to wheat flour [29] and rice flour [30,31,32] reduced the specific volume. Islam et al. [29] reported that this may be due to the baking suitability of soy flour. Although Sciarini et al. [44] observed an increase in the specific volume after the addition of 10% soybean flour to rice flour, when 20% was added, a negative effect was observed. Soybean protein could form a structure capable of incorporating more air bubbles and thus retain CO_2_ during mixing and proofing. Furthermore, soy proteins may interact with amylose and starch granules through non-covalent bonds, thereby reducing viscosity and interfering with the association of hydrogen bonds between the starch molecules [44]. Mizuhochikara alone, without any other ingredients, has a sufficient ability to produce bread of good quality; therefore, pregelatinized rice may not have any effect (Figure 7, second from the left). However, in the presence of soy, pregelatinized rice showed an improvement (Figure 7, second from the right). These results suggested that soy decreased the batter volume due to the disruption of the rice starch structure.

**Figure 8:**
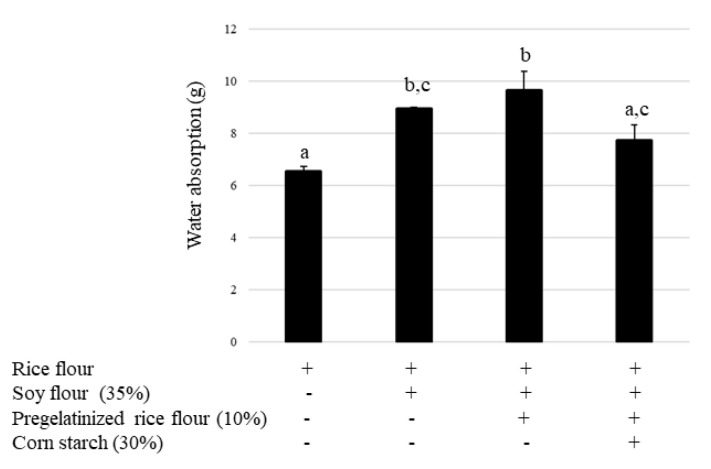
Effect of pregelatinized rice flour and cornstarch on water absorption (90 min). Data are shown as mean ± SD. Different letters indicate *p* < 0.05 between groups.

Bread quality is also affected by the water absorption of its constituent components. Accordingly, we examined whether there was a difference in water absorption between each bread type. In comparison with the control (rice flour alone), water absorption significantly increased in the soy bread and increased further by the use of pregelatinized rice. In contrast, the addition of cornstarch returned water absorption levels to those of the control (Figure 8).

**Figure 9:**
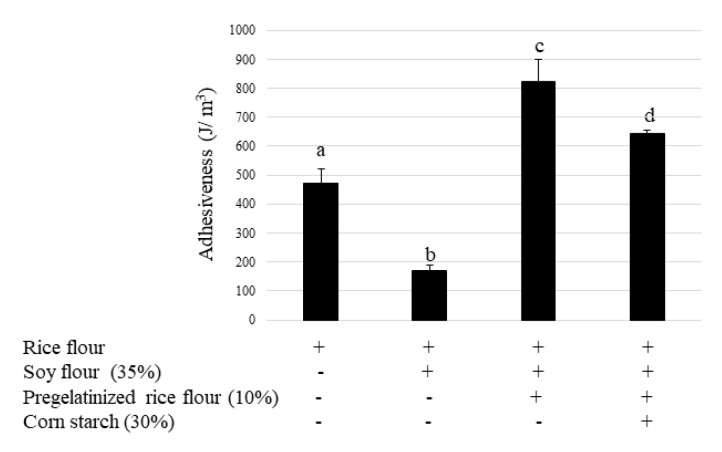
Effects of pregelatinized rice flour and cornstarch on adhesion of bread dough. Data are shown as mean ± SD. Different letters indicate *p* < 0.05 between groups.

Next, we examined whether water absorption influenced batter adhesion and whether pregelatinized rice flour increases the absorption of water, resulting in the thickening of the batter. Figure 9 illustrates how batter adhesiveness significantly decreased while using soy. Pregelatinized rice flour increased batter adhesiveness. These results indicate that pregelatinized rice flour allows the easy absorption of water, which influences batter viscosity and may lead to a retention of CO_2_ gas emitted during the fermentation process, thereby improving bread quality in terms of specific volume. Soy absorbs water but does not influence batter viscosity and only increases its weight; this may be due to the prevention of the pregelatinizing action of rice flour, thereby leading to a reduction in bread-specific volume. Cornstarch returned the increase in adhesiveness observed for pregelatinized rice flour to that of the control. Cornstarch suppressed water absorption and attenuated excess batter adhesiveness, resulting in an appropriate viscosity of the bread.

In summary, soy disrupted the cross-linking of rice starch (which determines adhesiveness) and interfered with the retention of CO_2_ gas in the batter, thus leading to a loss of specific volume in the bread.

**Figure 10:**
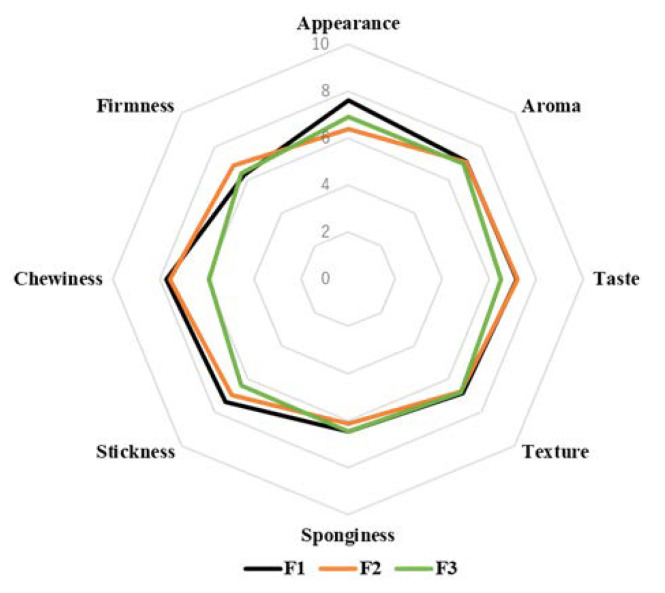
Sensory analysis of soy rice bread with preprocessed rice flour and/or cornstarch. Minimum score = 0 and maximum score = 10. F1: 35% replacement of soy flour (soy rice bread). F2: soy rice bread with preprocessed rice flour. F3: soy rice bread with preprocessed rice flour and cornstarch.

The results of the descriptive sensory analysis performed on the three breads are shown in Figure 10. Although no significant (*p* > 0.05) differences were observed between the 35% soy flour replacement bread (soy rice bread; F1), the soy rice bread with preprocessed rice flour (F2), and the soy rice bread with preprocessed rice flour and cornstarch (F3), the chewiness and stickiness of F3 tended to be lower than those of F1 or F2. Moreover, F3 tended to have sponginess and firmness qualities similar to those of F1, which were superior to those of F2. In the sensory evaluation, the following comments were made on the soy rice bread with preprocessed rice flour: “I felt that F2 is like a mochi, F3 has no bad taste and aroma, and F3 is dry as compared with F2”. It is important to note that we chose to include a panel of young people for this preliminary study to ensure safety, as textures similar to that of rice cake can readily cause dysphagia in elderly individuals. Thus, further sensory analysis is needed with a panel comprised of elderly people, and additional studies are required to assess the inhibitory effect of these breads on skeletal muscle declension.

An increasing number of people exhibit symptoms of gluten intolerance. The most common wheat flour substitutes for gluten-free bread are rice flour, which is mainly composed of carbohydrates and has a low protein content. Numerous studies have attempted to improve not only the quality but also the nutritive values of gluten-free bread by increasing its protein contents using plant- and animal-derived components. In this study, we evaluated the formulations for producing protein-rich bread. We used soy flour owing to its efficient protein synthesis activation and proteolysis inhibition effects. Although several studies have added soy flour to wheat or rice flour bread, the bread quality has remained uninvestigated. We observed a deterioration in the specific volume of soy bread; however, we attempted to improve the soy bread quality. Although there have been several studies on the addition of soybean flour to wheat and rice flour breads, improvements in bread quality have not been explored. We found that soybean inhibits the cross-linking of rice starch, which determines adhesion, and inhibits carbon dioxide retention in the batter, thereby reducing the specific volume of the bread. This can be applied to the production of gluten-free soy bread, for example, by preparing bread with improved cross-linking of rice starch, i.e., by either inhibiting or not inhibiting cross-linking. In this study, we used preprocessed rice flour to improve the cross-linking of rice starch, but we believe that this is possible with other materials as well, which are currently considering. Although we developed soy bread that may increase protein supply, it is necessary to continue investigating other formulations to improve nutritional aspects, quality, taste, and costs, including the inclusion of additional ingredients or changes to the recipe, while also confirming whether soy bread has beneficial effects on skeletal muscle in elderly people.

## 4. Conclusions

This study considers a nutritional approach to contribute to the prevention and improvement of skeletal muscle decline in the future. We attempted to produce quality soy bread as a breakfast meal option for the elderly, considering its provision of amino acids at an appropriate time to allow efficient muscle protein synthesis. Since the sustainability of use is a key factor to success, we considered bread taste and avoided gluten intolerance issues by using rice flour in analyzing bread quality. Nutritionally, one 70 g slice of soy bread delivered approximately 19% of the total BCAA and daily leucine requirements (60 kg body weight). In terms of baking quality, soy significantly decreased the specific volume of bread by inhibiting starch cross-linking that retains CO_2_ in the dough. However, the lost volume was recovered using preprocessed rice flour, and the taste was improved using cornstarch (which inhibited the excess of adhesiveness produced by preprocessed rice flour). We propose that soy bread has the potential to become an effective countermeasure against skeletal muscle declension. Soy bread is expected to enhance the nutritional value and quality of bread. Based on the findings, soy bread is a potential source of protein in the daily diet. However, further research is required to improve the nutritional aspects, baking quality, and cost of soy bread, while also ensuring optimal taste and minimal dysphagia in elderly individuals.

## Figures and Tables

**Table 1:** (**A**) Protein, amino acid contents in terms of BCAAs and lysine in 70 g of bread, and (**B**) recipes used in bread production.

(A)					
	Protein (g)	Leu. (mg)	Val. (mg)	Ile. (mg)	Lys. (mg)
Wheat bread	5.9	392	238	210	119
Rice bread	2.7	216	158	106	85
Soy bread	5.7	441	280	252	322
**(B)**					
		**Baker’ s % (*w/w*)**			
**Ingredient**		**Wheat**	**Rice**		**Soy**
Wheat flour		100	-		-
Rice flour		-	100		25
Soy flour		-	-		35
Preprocessed rice flour		-	-		10
Cornstarch		-	-		30
Water		68	82		100
Sugar		5	5		5
Salt		2.1	2.1		2.1
Dey yeast		1.5	1.5		1.5
Canola oil		5.7	5.7		5.7

## Data Availability

Data is contained within the article.
